# Thrombophilia in East Asian countries: are there any genetic differences in these countries?

**DOI:** 10.1186/s12959-016-0109-x

**Published:** 2016-10-04

**Authors:** Toshiyuki Miyata, Keiko Maruyama, Fumiaki Banno, Reiko Neki

**Affiliations:** 1 Department of Cerebrovascular Medicine, National Cerebral and Cardiovascular Center, Suita, 5658565 Japan; 2 Department of Molecular Pathogenesis, National Cerebral and Cardiovascular Center, Suita, 5658565 Japan; 3 Department of Food and Nutrition, Koriyama Women’s University, Koriyama, 9638503 Japan; 4 Division of Counseling for Medical Genetics, National Cerebral and Cardiovascular Center, Suita, 5658565 Japan; 5 Department of Perinatology and Gynecology, National Cerebral and Cardiovascular Center, Suita, 5658565 Japan

**Keywords:** East Asian, Genetic mutation, Pregnancy, Racial difference, Thromboembolism, Thrombophilia, Venous thrombosis

## Abstract

In recent years, genetic analyses of congenital deficiencies of three anticoagulant proteins, antithrombin, protein C (PC) and protein S (PS), in East Asian patients with venous thromboembolism (VTE) have greatly increased. The PS-K196E mutation is often identified in the Japanese population with an allelic frequency of 0.86 %, and a total of approximately 10,000 Japanese are estimated to be homozygotes. The heterozygotes show PS anticoagulant activities ranging from 40 to 110 %, and 16 % lower mean anticoagulant activity than that in wild-type individuals. Specific assay methods to identify carriers of this mutation have recently been developed. The mutation carriers are at risk of thrombosis during pregnancy but do not appear to be at risk for adverse pregnancy outcomes. To promote future research into this mutation and its relation to thrombosis, a thrombosis-prone mouse strain with the PS K196E mutation has been developed. We found the PS-K196E mutation and the heterozygous PS-deficiency in mice caused increased VTE, but did not cause aggravation of ischemic stroke, unlike factor V Leiden mutation. Importantly, the PS-K196E mutation is only identified in Japanese. This suggests that although East Asian populations including Japanese, Chinese, and Koreans are geographically and genetically close, the PS-K196E mutation seems to be Japanese-specific, suggesting that the mutation is a recent occurrence and fixed within the Japanese population. Some recurrent genetic mutations predisposing to VTE have been reported in Chinese and Korean populations. Although the genetic background for VTE is known to differ between populations with Caucasian descent and East Asian populations, some of the recurrent mutations differ even within the East Asian populations.

## Background

Venous thromboembolism (VTE) is a worldwide burden associated with death and disability. The R506Q mutation in factor V (FV), also known as the FV Leiden (FVL) mutation, is well known as an established genetic risk factor for VTE in individuals of Caucasian descent [1, 2]. The FVL mutation was identified in 1994; in 1995 we attempted to identify the FVL mutation in the Japanese population, but failed due to its absence in East Asian populations [3]. To identify the genetic variants of VTE in the Japanese population, we performed a nationwide collaborative study of 5 hospitals supported by the Ministry of Health, Labour and Welfare of Japan. In this study, we genotyped the 5 genetic variations and found that a Lys196-to-Glu substitution in the protein S (PS) gene (PS-K196E, rs 121918474, c.586A>G, also known as PS Tokushima, PS-K155E in the mature protein numbering) was a genetic risk factor for VTE with an odds ratio of 4.72 [4, 5]. This finding was published in 2006.

Historically, the PS-K196E mutation was identified simultaneously in 1993 and 1994 by two independent Japanese groups in families with or without VTE [6, 7]. Individuals with the PS-K196E mutation in families without VTE showed PS anticoagulant activity within a normal limit, suggesting the mutation was a neutral polymorphism [6], whereas individuals with the mutation in another family with VTE showed low PS anticoagulant activity, suggesting PS-K196E was causative for VTE [7, 8]. Therefore, whether the mutation would be a risk for VTE was debatable. A subsequent our study, however, revealed that both observations were correct, because the heterozygous carriers of this mutation in a general population showed anticoagulant activities ranging from 40 up to 110 %, that were substantially overlapped with those of wild-type individuals [9]. In this short review, we will summarize the history of the PS-K196E mutation and discuss the similarities or differences of the genetic background for VTE among East Asians.

## Review

### Prevalence and unique geographical profile of PS-K196E mutation

Three case-control studies of VTE independently indicated that the PS-K196E mutation is a genetic risk factor for VTE in the Japanese population (odds ratio = 3.74–8.56) (Table 1) [4, 10, 11]. In the Japanese population, a total of 81 heterozygotes have so far been identified among 4686 individuals enrolled in four studies [4–6, 10, 11] and two public genome databases ([12], http://www.genome.med.kyoto-u.ac.jp/SnpDB/index.html), yielding an allele frequency of 0.86 % for the PS-K196E mutation, and indicating that approximately 1 of 58 Japanese is a heterozygous carrier. We thus estimate that approximately 1 of every 13,000 Japanese individuals is homozygous for the mutant allele. The Japanese population was approximately126 million on 1 January 2015, meaning that as many as 9440 individuals are homozygotes for the PS-K196E mutation. Thus, a substantial number of Japanese carry the mutant PS 196E allele, as a heterozygote or homozygote, and are likely at risk of developing VTE. So far, no homozygotes have been identified in the general population, but three homozygotes have been found among 258 VTE patients, yielding an incidence of 1.2 % among patients with VTE [10, 13].

**Table 1:** Influence of genetic variants on VTE in protein S, protein C, and antithrombin genes in East Asian population

Gene name: Nucleotide change	Amino acid change	No. of deficiency/total (%)		Odds ratio (95 % CI)	Population	Reference
		VTE	Controls			
Protein S						
c.586A>G	K196E	5/85 (5.8 %)	5/304 (1.6 %)	3.74 (1.06–13.2)	Japanese	Kinoshita et al., 2005 [10]
c.586A>G	K196E	15/161 (9.3 %)	66/3651 (1.8 %)	5.58 (3.11–10.01)	Japanese	Kimura et al., 2006 [4]
c.586A>G	K196E	6/60 (10.0 %)	3/234 (1.3 %)	8.56 (2.07–35.30)	Japanese	Ikejiri et al., 2010 [11]
Protein C						
c.565C>T	R189W	5/116 (4.3 %)	11/1292 (0.9 %)	5.10 (1.70–14.8)	Chinese	Tsay et al., 2004 [26]
c.565C>T	R189W	59/1003 (5.9 %)	9/1031 (0.9 %)	7.10 (3.50–14.39)	Chinese	Tang et al., 2012 [27]
c.574_576del	K193del	68/1003 (6.8 %)	25/1031 (2.4 %)	2.93 (1.84–4.67)	Chinese	Tang et al., 2012 [28]
c.631C>T	R211W	14/500^a^ (2.8 %)	6/2953^b^ (0.2 %)	n.d.	Korean	Kim et al., 2014 [16]
c.1218G>A	M406I	9/500^a^ (1.8 %)	0/2953^b^ (0.0 %)	n.d.	Korean	Kim et al., 2014 [16]
Antithrombin						
c.235C>T	R79C	2/500^a^ (0.4 %)	7/3046^b^ (0.2 %)	n.d.	Korean	Kim et al., 2014 [16]
c.442T>C	S148P	1/500^a^ (0.2 %)	5/3046^b^ (0.2 %)	n.d.	Korean	Kim et al., 2014 [16]

^a^Genetic analysis was performed in VTE patients with anticoagulant activity of less than 2.5 percentile of the local reference intervals. ^b^Genetic analysis was performed in population individuals with anticoagulant activity of less than 1 percentile of the population. *n.d*. not determined

The risk of developing VTE conferred by PS-K196E thus appears to be fairly mild (odds ratio: 3.74–8.56). Accordingly, some VTE patients have been found to bear PS-K196E along with other deleterious genetic mutations, such as PC-K193del, PC-R221W, and PC-V339M, indicating that carriage of the PS-K196E mutation concomitant with other predisposing mutations for thrombosis facilitates the onset of VTE [13].

Although East Asians, including Japanese, Chinese, and Koreans, are geographically and genetically close, the PS-K196E mutation has so far been identified only in Japanese and not in Chinese and Koreans, even in the VTE patients [14–17]. Thus, PS-K196E mutation seems to be Japanese-specific, suggesting that the mutation is a recent occurrence and fixed within the Japanese population.

### Anticoagulant activity of heterozygous carriers of PS-K196E mutation

We performed a phenotype-genotype analysis in individuals with or without PS-K196E mutation in the Japanese general population (*n* = 1862), and found that heterozygotes (*n* = 34) showed anticoagulant activities ranging from 40 to 110 %, and the anticoagulant activities between heterozygotes and wild-types were substantially overlapped [9]. Thus, the anticoagulant activity assay widely used for the identification of PS deficiency is not a good tool to identify the PS-K196E carriers. The mean PS anticoagulant activity in heterozygotes, however, was 16 % lower than that in wild-type individuals. Acquired PS deficiency is associated with nephrotic syndrome or pregnancy. These conditions may further promote the PS-activity reduction in mutation carriers, thereby enhancing the risk for VTE.

### DVT and adverse pregnancy outcome in pregnant women with PS-K196E mutation

The PS-K196E mutation appears to be a genetic risk factor for deep vein thrombosis (DVT) during pregnancy [18]. Among 18 patients with DVT during pregnancy and the postpartum period, we identified 4 DVT patients with PS mutation and 1 DVT patient with PC mutation [18]. Among the 4 DVT patients with PS mutation, 2 were heterozygous for the PS-K196E mutation with DVT onset of 27 and 10 weeks, respectively. None carried mutations in the antithrombin gene. All patients with the genetic mutation showed DVT at the first or second trimester and none showed DVT at the third trimester or postpartum period. It is well known that the PS anticoagulant activity is decreased in pregnancy. Since the genetic mutation accelerates DVT onset, DVT onset at the early stage of pregnancy in patients with the PS-K196E mutation would be reasonable.

Adequate maternal-fetal circulation is important for a successful pregnancy outcome, and disturbance of circulation may result in serious adverse pregnancy outcomes, such as severe preeclampsia, placental abruption, intrauterine fetal death (IUFD), or severe fetal growth restriction (FGR). The causes of these adverse pregnancy outcomes are not known, but they may be associated with abnormal placental vasculature and disturbances of hemostasis.

In a previous report, we tried to address whether adverse pregnancy outcomes in Japanese parturients are associated with the PS-K106E mutation or rare genetic mutations in three anticoagulant genes: PS, PC, and antithrombin [19]. We enrolled 330 Japanese patients with adverse pregnancy outcomes and divided them into 233 patients with two or more miscarriages and 114 patients with FGR and/or IUFD; 17 patients belonged to both groups. In this cohort, the PS-K196E mutation was identified in 4 out of 233 patients with two or more miscarriages and 2 out of 114 patients with FGR and/or IUFD. The frequencies of the PS-K196E mutation in these patient groups were not different from that in the Japanese general population. Therefore, the PS-K196E mutation does not confer a significantly increased risk of these adverse pregnancy outcomes. Our results were compatible to those of a recent large prospective cohort study in Canada in which carriage of FVL or the prothrombin G20210A mutation did not increase the risk for the adverse pregnancy outcomes of pregnancy loss, small for gestational age, preeclampsia, or placental abruption [20].

In our study, rare nonsynonymous mutations in the three anticoagulant genes were found in only 3.3 % (11 out of 330) of patients with adverse pregnancy outcomes, indicating that carriers with rare mutations were not dominant in patients [19]. Therefore, although the small fraction of patients with adverse pregnancy outcomes may be explained by the rare variations in the three anticoagulant genes, these rare mutations are not likely a major cause of the development of adverse pregnancy outcomes.

### Screening methods for PS-K196E mutation carriers

As described above, the anticoagulant PS activity is not a reliable measure for the identification of PS-K196E mutation carriers, and thus until recently the only accurate detection assay was DNA analyses such as direct sequence analysis, restriction fragment length polymorphism analysis, or the TaqMan genotype discrimination method. More recently, however, two plasma assay methods were developed to distinguish the carriers from the wild-type individuals.

We developed monoclonal antibodies specific to the PS K196E mutant using GANP transgenic mice that show an increased frequency of somatic mutations in the Ig-variable region and are suitable for high-affinity antibody production [21]. We constructed a sandwich enzyme-liked immunosorbent assay (ELISA) using one of the monoclonal antibodies for detecting plasma samples of individuals with the PS K196E mutation [21]. This assay is a simple, efficient, and accurate tool for the detection of PS-K196E carriers. The ELISA assay can detect the PS K196E mutant in plasma and can be identified in carriers even though they are under warfarin treatment or during pregnancy.

Another assay has been developed using the specific activity of PS for the discrimination of heterozygotes from the wild-types [22]. Since individuals with PS type II deficiency who are carriers of the PS-K196E mutation show the reduced PS activity with normal antigen limits, the specific activity obtained as the ratio of the activity level divided by the antigen level would be reduced to around 0.5. Actually, the mean PS specific activity of the PS-K196E heterozygote carriers was 0.64 [22]. This assay can distinguish individuals with the PS type II deficiency who are carriers of the PS-K196E mutation from wild-type individuals. However, individuals with low specific activity do not always have the PS K196E mutation and may have other missense mutations.

### Mice bearing PS-K196E mutation

Guidelines for establishing pathogenic causality for rare genetic variants have recently been published [23]. They suggest that the strongest evidence for causality comes from disruption of the candidate gene in a model organism which recapitulates the pathology in humans. To evaluate the thrombogenicity of the PS-K196E mutation, we generated PS-K196E knock-in mice and analyzed their phenotypes in comparison with heterozygous PS-deficient mice and factor V-R504Q knock-in mice (FVL mice) [24]. Plasma from PS-K196E homozygous mice had 67 % of normal APC cofactor activity, similar to a purified recombinant murine PS-K196E mutant that had 49 to 60 % of normal PS activity. Mouse C4BP does not contain the PS-binding ß-chain, which is a pseudogene in mice, so interpretation of the APC cofactor activity is uncomplicated by C4BP considerations.

The susceptibility of PS-K196E mice to VTE was assessed in multiple models, including (i) an electrolytic inferior vena cava model of venous thrombosis that produces a non-occlusive and consistent thrombus in the presence of constant blood flow, (ii) a tissue factor-initiated pulmonary thromboembolism model, and (iii) a polyphosphate-initiated pulmonary thromboembolism model [24]. All thrombotic parameters in these thrombosis models, including mortality, showed that the PS-K196E mutation in mice caused increased VTE, very similar to the heterozygotes for PS deficiency and to the murine FVL mutation. Heterozygotes for the PS-K196E mutation gave a milder thrombotic phenotype than homozygotes. These results demonstrate a causal link between the PS-K196E mutation and thrombophilia, strongly supporting the notion that the PS-K196E mutation is a human genetic risk factor for VTE. PS K196E mice would be valuable for disease-related studies in which FVL mice have been examined.

Currently, the extent to which the PS-K196E mutation or PS deficiency are risk factors for arterial occlusive diseases is not clear. To explore the arterial occlusive risk of these PS genetic variations, we used a cerebral focal ischemia-reperfusion model. We found no exacerbation of ischemic stroke in PS-K196E mice and heterozygous PS-deficient mice [24]. We previously observed that FVL mice showed increased infarct volumes compared with wild-type mice [25]. Our studies suggest that PS-K196E mutation in mice does not cause aggravation of ischemic stroke, unlike factor V Leiden mutation.

### Thrombophilic mutations in Chinese and Korean populations

Genetic analyses in patients with VTE and in the general populations have been reported from China and Korea as well. The PC Arg189Trp mutation (PC-R189W, c.565C>T, PC-R147W in the mature protein numbering) and the PC Lys193del mutation (PC-K193del, c.574-576del, PC-K151del in the mature protein numbering) were reported to be risk factors for VTE in the Chinese population, with odds ratios of 5.10–7.30 and 2.93, respectively (Table 1) [26–28]. Both R189 and K193 residues are located on a 21-amino-acid peptide, with 8 basic Lys and Arg amino acids on it, in the C-terminal part of the PC light chain. The PC-R189W and K193del mutations are present in 0.9 and 2.4 % of the Chinese population, respectively [26, 28]. The former mutation was not identified in 767 Japanese, but the later was identified in 10 out of 767 Japanese including one homozygote [http://www.genome.med.kyoto-u.ac.jp/SnpDB/index.html], and both mutations were identified in Japanese and Korean patients with VTE. Both heterozygotes are associated with reduced anticoagulant activity and a relatively normal PC antigen level, indicating type II deficiency. The recombinant PC-R189W mutant shows normal amidolytic and proteolytic activities in the absence of cofactors, but exhibits about 3 times lower affinity for binding to EPCR [29]. The recombinant PC-K193del mutant has approximately 2–3 fold impaired anticoagulant activity in the presence of PS [29]. Individuals with the PC-K193del mutation show normal amidolytic activity of PC but reduced anticoagulant activity [13, 30].

In the genetic analysis in the Korean population, the PC Arg211Trp (PC-R211W, PC-R169W in the mature protein numbering) and Met406Ile (PC-M406I, PC-M364I in the mature protein numbering) mutations, both of which cause type I PC deficiency, were identified in 2.8 and 1.8 % of the VTE patients, and 0.2 and 0 % of the Korean population (Table 1), respectively [16]. Both PC mutations have been identified in Japanese VTE patients [30], but not in the Japanese Human Genetic Variation Database [http://www.genome.med.kyoto-u.ac.jp/SnpDB/index.html]. In the antithrombin gene, the Arg79Cys and Ser158Pro mutations (AT-R79C and AT-S158P, AT-R47C and AT-S116P in the mature protein numbering, respectively) have been identified in 0.4 and 0.2 % of Korean VTE patients, respectively (Table 1). Both mutations have previously been identified in Japanese VTE patients with heparin-binding defects. Since both were also present in the Korean general population, they would confer weak risk for VTE (Table 1), if any. We previously demonstrated that the antithrombin deficiency with the heparin-binding defects conferred a lower VTE risk than type I deficiency [31], and this finding was compatible with the Korean VTE study.

## Conclusion

We identified a PS-K196E mutation as a mild genetic risk factor for VTE. The mutation is specific to the Japanese population and not present in Chinese and Koreans. We generated a mouse line carrying the PS K196E mutation. The thrombosis phenotypes observed in the PS K196E mice indicated a strong link between the PS-K196E mutation and thrombosis. Compared with the frequencies of genetic mutations among East Asian populations, many genetic mutations predisposing to VTE, except for the PS K196E mutation, are commonly distributed, but their frequencies differ among East Asian countries.
